# The overlooked greatwall: a new perspective on mitotic control

**DOI:** 10.1098/rsob.120023

**Published:** 2012-03

**Authors:** David M. Glover

**Affiliations:** Department of Genetics, University of Cambridge, Downing Street, Cambridge CB3 9JW, UK

**Keywords:** mitosis, Greatwall kinase, Endos, PP2A

## Abstract

The role of the dual specificity protein phosphatase, Cdc25, in activating the cyclin-dependent kinase-cyclin B complex (Cdk1-CycB) by overcoming the inhibitory Wee1 kinase is a long-established principle for mitotic entry. Recently, however, evidence has emerged of a regulatory network that facilitates Cdk1-CycB activity by inhibiting the form of protein phosphatase 2A having a B55 regulatory subunit (PP2A-B55). Here, I review the genetic and biochemical evidence for Greatwall kinase and its substrate Endosulphine as the key components of this previously obscure regulatory network. Not only is the inhibition of PP2A-B55 by phospho-endosulphine required to prevent dephosphorylation of Cdk1-CycB substrates until mitotic exit, but it is also required to promote Cdc25 activity and inhibit Wee1 at mitotic entry. I discuss how these alternating states of preferential PP2A-B55 or Cdk1-CycB activity can have an impact upon the regulation of Polo kinase and its ability to bind different partner proteins as mitosis progresses.

## 2. A short history of the protein kinases regulating mitosis

In this paper, I would like to review some recent studies of the regulation of protein dephosphorylation as a counter to the activity of the Cdk1 mitotic kinase. I will try and place some of these recent findings into a historical context in order to view the broader roles of these proteins in mitotic regulation. This recent work, like many of the pioneering studies of the cell cycle, has relied upon embryos that undertake rapid cleavage divisions. Such embryos, from insects, echinoderms, molluscs and amphibians, have been good friends of the cell cycle research community because they all come fully loaded with a maternal dowry of proteins needed for the cleavage division cycles, thus making them amenable for biochemical and, in some cases, genetic studies. Much has been written about such biochemical approaches that have their origins in the work of Yoshio Masui & Clement Markert [1] and James Reynhout & Dennis Smith [2] of maturation promoting factor (MPF), a cytoplasmic entity that could be withdrawn from unfertilized frog eggs and injected into oocytes, causing them to ‘mature’ (i.e. undertake meiosis I and arrest in meiosis II). An entity with similar properties was soon found to be active in the starfish oocyte by Takeo Kishimoto & Haruo Kanatani [3], where the involvement of increased protein kinase activity in meiotic maturation was quickly appreciated by Marcel Doree and co-workers [4]. Together Fred Lohka & Yoshio Masui established a cell-free system from activated frog eggs in which sperm nuclei would undergo decondensation and DNA synthesis, and condense to form mitotic chromosomes [5]. This, together with work from Marc Kirschner's laboratory in the early 1980s, was instrumental in developing *Xenopus* embryos as a system in which to study the oscillations of MPF activity in the cleavage cycles that are dependent on protein synthesis [6]. Over the intervening decades, we have realized what a fantastic system this is, not only in providing cell-free extracts in which the oscillatory behaviour of MPF activity could be examined in the test tube, but also in providing an *in vitro* system that is able to recapitulate the complex dynamics of spindle formation and function. However, it was the cleavage embryos of sea-urchins that allowed Tim Hunt and co-workers to first observe the mitotic cyclins, proteins undergoing periodic cycles of destruction and re-synthesis in phase with mitotic progression [7]. Of course, all eventually became crystal clear when Fred Lohka, Marianne Hayes and Jim Maller got to work to purify MPF from *Xenopus* [8]. MPF proved to be a complex of a mitotic cyclin and a protein kinase, now known as Cdk1, whose genes, *CDC28* and *cdc2^+^*, had been described in the respective budding and fission yeasts by Lee Hartwell [9] and Paul Nurse [10]. Nurse's work revealed Cdc2 kinase's conserved function in mitotic entry [11] and also identified the gene for its activating protein Cdc25p [12], later discovered to be a dual-specificity protein phosphatase. The protein kinase opposing Cdc25p, which phosphorylates a critical tyrosine residue in Cdc2p's active site to inhibit the kinase, was the product of the *wee1^+^* gene [13]. This regulatory wiring turned out to be shared by all eukaryotic organisms. Collectively, therefore, these findings provided the field with an explosive burst of activity in the late 1980s that established the key facets of mitotic regulation and a broad understanding of how the conserved mitotic kinase, Cdk1, was regulated. As we shall see in this review, we can now add a new dimension to this regulatory process.

**Figure 1.**
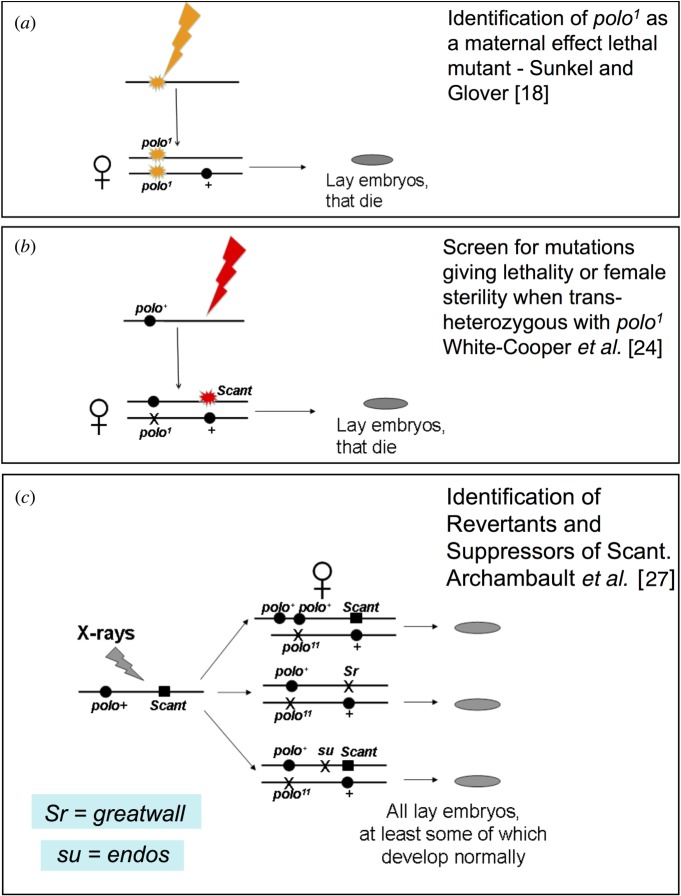
Origins of some of the alleles of *polo* and *greatwall*. (*a*) The orginal *polo* allele was identified as a mutation that, when homozygous in the mother, led to mitotic abnormalities and death of syncytial *Drosophila* embryos. *Scant* was isolated as a mutation that, when trans-heterozygous with *polo* (one mutant copy of *Scant* and one mutant copy of *polo*), caused females to produce embryos that died owing to a specific mitotic defect—loss of centrosomes from one pole (see text and figure 2). Scheme for the identification of mutations that would suppress the + *Scant/polo* + *phenotype*. These were *polo^+^* duplications; revertants of *Scant* to its recessive alleles, *Sr*; and a second-site suppressor, *su. Sr* was identified as *greatwall, su* as *endos.* We may also refer to *Scant* as *gwl*^Scant^.

Genetic studies of cell cycle regulation in *Drosophila* also got under way in the 1980s. However, by and large, the fly community followed independent genetic routes that lagged a little behind their yeast colleagues in identifying mitotic regulatory genes. These exploited the fact that two stages of the *Drosophila* life cycle are particularly dependent on the cell division cycle: the embryo, whose early cycles are driven by maternally supplied proteins; and the late larval/pupal stages, when the imaginal tissues and central nervous system proliferate to form the adult structures. Because much of the earlier stages of larval development involves the growth of tissue whose cells undergo endoreduplication, repeated rounds of S-phase in the absence of mitosis, there is little demand for the mitotic machinery during these stages. Consequently, the maternal provision of many wild-type proteins from a heterozygous mutant mother is sufficient to allow her homozygous mutant offspring to survive to third instar larval or pupal stages. Mitotic defects then begin to accumulate in the proliferating diploid tissues that will form the adult structures. Nevertheless, for some gene products, the transition from maternal to zygotic provision occurs very much earlier—in the embryo at cycle 14 immediately after cellularization of the nuclei of the synctium. Bruce Edgar & Pat O'Farrell [14] showed that one such gene that has to be expressed zygotically at this stage is *string,* which encodes one of the two *Drosophila cdc25* homologues. It was Maurizio Gatti & Bruce Baker [15], however, who classically exploited the transition between maternal and zygotic control of cell division by screening mutants exhibiting late larval/pupal lethality in a search for genes required for cell proliferation at these stages. Roger Karess in my laboratory followed their example by carrying out one of the first P-transposon screens for such mutants that was instrumental in defining several of our favourite genes. However, we also adopted a complementary genetic approach to study the maternal contribution to cell cycle regulation. Encouraged by Janni Nuesslein-Volhard, we first screened Janni's own collection of *Drosophila* maternal effect lethal mutants generated by EMS mutagenesis. Notably, this led to the characterization of *gnu* [16], a gene now known to participate in regulating the translation of maternal mRNA for Cyclin B [17]. Strikingly, Janni's collection harboured mutant genes for two interesting protein kinases, which we named *polo* [18] (figure 1*a*) and *aurora* [19]. We chose these names because their phenotypes, defective spindle poles, reminded us of phenomena at the geomagnetic poles of the Earth. Although these protein kinases were in the shade of Cdk1-cycB for many years, we now know that they have key roles in mitotic progression and these have been reviewed elsewhere [20,21]. Polo, which will later become a lead character in this essay, plays multiple roles in co-ordinating mitotic progression. In so doing, it moves about the cell from centrosomes to kinetochores, and finally to the central spindle, to act out its part in a multitude of processes from centrosome maturation to cytokinesis.

## 3. The genetical trail: from Arctic to the Greatwall via the Antarctic

**Figure 2.**
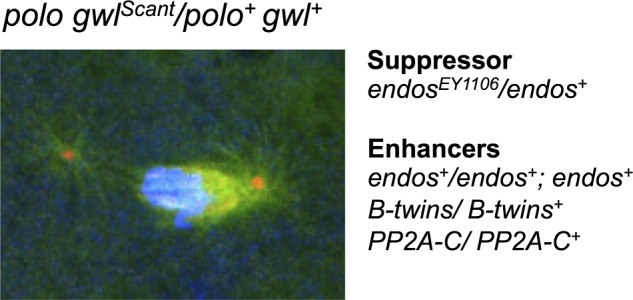
The ‘*Scant*’ phenotype; its suppressor and enhancers. Typical mitotic figure from embryos derived from a mother with one mutant copy of *polo* and one mutant copy of *gwl*^Scant^. We first named the gene *Scant* after Scott of the Antarctic, the British explorer who set out to find the mysterious southern geomagnetic pole of the Earth in a ship, the Discovery, which is now anchored in full view of the University of Dundee campus. Reducing the wild-type gene dosage of *endos* in the mother suppresses, whereas increasing the *endos* gene dosage enhances the phenotype.

It was to be genetic studies with *polo* that set our group on a trail to the then-unknown destination of Greatwall kinase and its substrate Endos. This began in the late 1980s, when we had just discovered the *polo* gene and were still unaware that it encoded a protein kinase. We drew inspiration from the ideas of Minx Fuller that second-site mutations that failed to complement mutations in the male-specific tubulin gene represented genes encoding interacting proteins within protein complexes [22] (box 1). This led Tano Gonzalez and myself to write a grant application in which we proposed to take the *polo^1^* mutation, a hypomorphic allele with reduced activity, together with mutations in several other mitotic genes and screen for non-complementing mutations at other sites. We got the grant and I am sure we somehow put it to good use, but the project was not brought to fruition until Helen White-Cooper took on the project for her PhD studies in the early 1990s [23]. Helen actually recombined five mitotic mutants onto the same chromosome and carried out a screen to isolate non-complementing mutations following EMS mutagenesis [24]. One of these five genes was represented by the *polo^1^* allele, and indeed one of many new mutants discovered failed to complement *polo* in this test (figure 1*b*). This turned out to be the first allele of *greatwall*; it was not a straightforward recessive allele but rather a gene with a dominant phenotype that only became apparent when the levels of functional Polo were reduced, and then only in the embryos derived from mothers of such a genotype. We named the gene *Scant* (after **Sc**ott of the **Ant**arctic) because of its mutant phenoptype, which led us to suspect that the gene would be interesting (see figure 2 and legend): embryos derived from *polo^1^+/+Scant* mothers developed mitotic spindles from which centrosomes were lost from one of the poles. When this first happened, most centrosomes popped right back into place later in the cycle, but eventually the outcome was mitotic mayhem and embryonic lethality.

Box 1. **Second-site non-complementing mutations.**

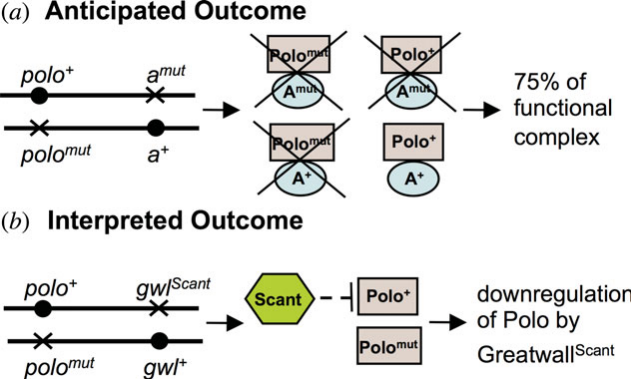

(*a*) Non-complementing second-site mutations lead to mutant phenotypes when the two genes under study are each present as one wild-type copy and one mutant copy. The resulting mutant phenotype can be accounted for in a number of ways. In this example, it is imagined that the second-site gene encodes a partner of Polo kinase essential for its function as a heterodimer and that both mutant genes carry recessive loss-of-function mutations. Thus, of the four possible combinations of heterodimeric protein that can be formed between wild-type and mutant proteins, only one will be functional. This 75 per cent reduction of functional heterodimer can lead to a mutant phenotype.

(*b*) The *Scant* mutation, identifying the first mutant allele of *greatwall*, was identified as a second-site non-complementing mutation of *polo^1^*, but it is a gain-of-function mutant that we now know to encode a hyperactive (i.e. gain-of-function) form of Greatwall kinase. Thus, *Scant* exerts a dominant mitotic phenotype in the presence of mutations showing reduced Polo activity. This repressive effect of *greatwall*^Scant^ upon *polo* is discussed in the text.

Recessive alleles of *greatwall* were later to be isolated by Mike Goldberg's group, who gave the gene its popular name [25]. These recessive mutations resulted in cell cycle delay at the G2-to-M transition and mitotic chromosomes showing unusual states of condensation. Contemporaneously, we had found Greatwall kinase through an RNAi screen to downregulate every protein kinase in the *Drosophila* genome in cultured *Drosophila* cells, in a search for protein kinases regulating cell cycle progression [26]. It was some time before we recognized *greatwall* as an allele of *Scant.* This was really the result of Adelaide Carpenter's inspiration to set up a screen to identify *Scant* revertants (figure 1*c*). As *polo^1^ +/+ Scant* mothers produce no viable offspring, it was relatively straightforward to mutagenize chromosomes carrying *Scant,* this time by X-irradiation, place the mutagenized chromosome against one carrying *polo^1^* and search for mothers who produced viable progeny. At last, in the mid-2000s, we had isolated several *Scant* revertants and grouped them into an allelic series of *greatwall* mutations [27]. An amorphic allele displayed pupal lethality, indicating that, in common with many other cell cycle genes of *Drosophila*, it was essential for the proliferation of diploid tissues to form the adult. Only once we had these recessive alleles were we able to map the *Scant* locus using a combination of classical and molecular genetic approaches, and identify it on the genome as encoding the Greatwall kinase. We found that the *Scant* mutation corresponded to a single amino acid change, K97M, which we could show, when introduced into Greatwall kinase to be expressed in cultured cells, resulted in dramatically increased activity towards artificial substrates.

Depletion of Greatwall from cultured cells led to mitotic delays and a characteristic phenotype of conjoined chromatids scattered upon mitotic spindles that were elongated as if in anaphase B [26]. Of our own recessive *gwl* alleles isolated as *Scant* revertants, several showed mutant phenotypes in larval neuroblasts similar to those previously described by the Goldberg laboratory, and one allele, a female-specific germ-line splicing mutant, showed only female sterility [27]. It turned out that flies have two isoforms of Greatwall, one of which is the only form produced in the female germ-line and is absolutely essential for normal progression through female meiosis. The oocytes of females hemizygous for this allele, *gwl^Sr18^,* fail to arrest in metaphase of meiosis I, and both homologues and sister chromatids separate on elongated, often highly branched meiotic spindles.

Our screen for *Scant* revertants also produced two other interesting types of mutant (figure 1*c*). The first of these were duplications of the *polo* locus that rescued the maternal effect lethality by restoring *polo* to the wild-type gene dosage. This was in accord with the need for reduced *polo* function in order to see reduced fertility in the presence of *gwl^Scant^*. The second was exemplified by a ‘third site’ suppressor of *polo^1^ +/+ Scant* that turned out to be a mutation in the gene for the Greatwall kinase substrate Endos [28].

*endos* encodes a small phospho-protein, α-endosulphine, originally (but probably erroneously) suggested in vertebrates to be the ligand of sulphonylurea receptor K^+^ channels [29]. *Drosophila endos* mutants showed female sterility [30], later shown to be due to a failure of oocytes to progress properly to metaphase I and subsequently undertake aberrant meiosis [31]. The meiotic phenotype was said to resemble that of females mutant for the *Drosophila* germ-line-specific form of Cdc25, *twine* [32–34], and, interestingly, levels of both Twine and Polo kinase proteins were reported to be reduced. However, the latter observation is curious because genetic experiments predict that Greatwall/Endos should antagonize Polo function (see below). Indeed why specific protein levels should be reduced is not clear because, as we shall see below, the majority of the mutant phenotypes of *endos* can be accounted for by the ability of its phosphorylated form to inhibit PP2A with a B55 regulatory subunit.

Subsequently, we have found mutations in a number of other cell cycle regulatory genes that either suppress or enhance *polo^1^ gwl^Scant +^* (figure 2). Of these, we focused upon genes for subunits of PP2A because of the findings from biochemical studies that this phosphatase was regulated by Greatwall kinase (see below) and because of our earlier findings in flies that the regulatory B subunit of PP2A encoded by *twins/abnormal anaphase resolved* (*aar*) was required for anaphase progression, and this form of PP2A preferentially dephosphorylated substrates of Cdk1-cycB [35–37]. Moreover, a separate screen carried out by Vince Archambault and his co-workers also identified mutations in PP2A as enhancers of either a strong hypomorphic *polo* mutant or of *Scant* [38]. Our study showed that lowering the dosage of *endos* suppressed *polo^1^ gwl^Scant^*, allowing many embryos to survive [28]. In contrast, lowering the dosage of either the catalytic C subunit or the B55/Twins regulatory subunit of PP2A enhanced the maternal-dominant effect of *polo^1^ gwl^Scant^* (figure 2). Increasing the gene dosage of wild-type *endos* also acted as an enhancer. Thus, Endos and PP2A-twins appeared to be acting antagonistically in this genetic test.

**Figure 3.**
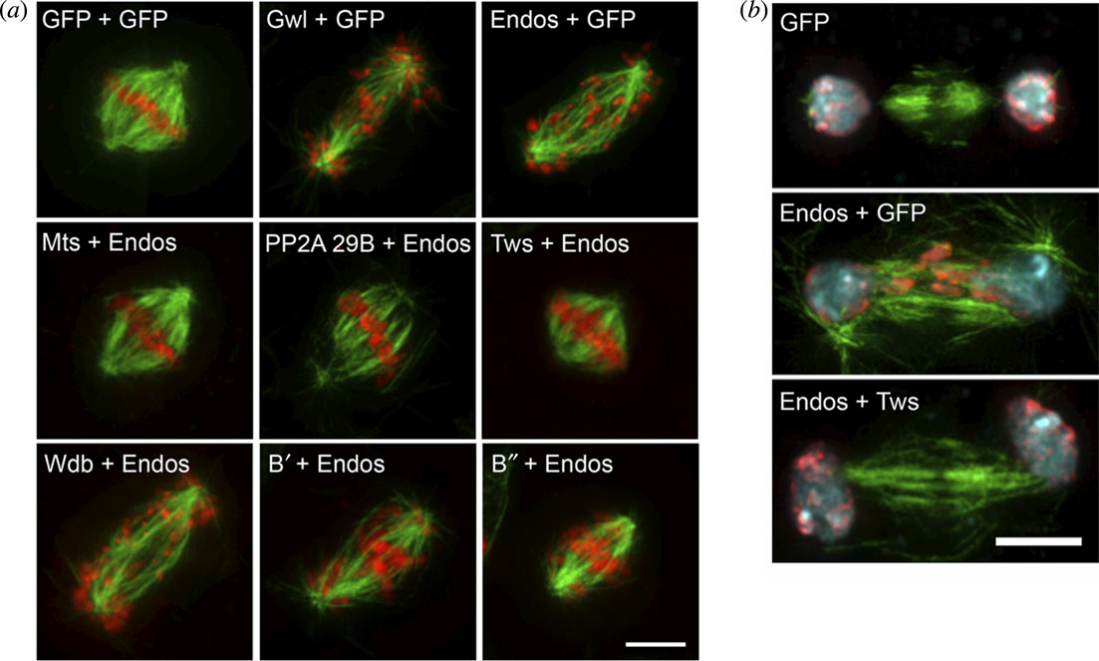
Suppression of the *endos* knockdown phenotype in cultured cells by simultaneous knockdown of *PP2A-B55*^twins^. (*a*) RNAi-mediated depletion of either Greatwall or Endos results in prolonged mitoses in which chromosomes remain scattered on elongated spindles before attempting anaphase. This phenotype is suppressed by the simultaneous depletion of either the catalytic subunit of PP2A (encoded by *mts—microtubule star*), the structural A subunit (encoded by *PP2A 29B*) or the B subunit (encoded by *twins* also known as *aar-abnormal anaphase resolved*). It is not suppressed by knocking down the three other regulatory B subunits in *Drosophila* (*wdb,widerborst*; *B′*; or *B″*). (*b*) Cells depleted of Endos display lagging chromosomes at anaphase. This phenotype is rescued by simultaneous depletion of the PP2A B-subunit, Twins.

A second set of experiments using RNAi to knockdown gene function in cultured *Drosophila* cells also suggested that Endos was required to antagonize the function of PP2A-B55^Twins^ [28]. The effects of either *endos* or *greatwall* knockdown in cultured *Drosophila* cells are very similar; mitotic progression into anaphase is delayed and in fixed preparations scattered chromosomes are seen on unusually elongated spindles (figure 3*a*). The phenotype of the double knockdown was not significantly more severe, suggesting that the two genes work in the same pathway. The *endos* knockdown phenotypes could be suppressed by co-depleting the catalytic (C), structural (A) or the regulatory B55-subunit of PP2A encoded by *twins.* There are genes for four different regulatory B subunits of PP2A in the *Drosophila* genome, and co-depletion of the other three, Widerborst, B′ or B″, had no effect*.* The phenotype of lagging and bridging anaphase chromosomes in *endos* RNAi-treated cells could also be suppressed by knocking down PP2A-B^twins^ (figure 3*b*). Thus, Endos appeared to counteract PP2A-B55^Twins^ functions; a finding entirely consistent with parallel biochemical studies in *Xenopus* that I shall now review. These showed that inhibition or depletion of PP2A-B55 from mitotic extracts rescues the inability of Gwl-depleted extracts to enter M phase [39,40] and that Endos becomes a potent inhibitor of PP2A after phosphorylation by Greatwall [41,42].

## 4. The biochemical trail: do not fantasize about the poles, stay in the coldroom

After identifying the first recessive *greatwall* alleles in *Drosophila,* Mike Goldberg returned to his biochemical roots and switched to studying the *Xenopus* counterpart of Greatwall kinase [43]. His group showed the kinase was activated in mitosis, probably by Cdk1, and that the depletion of Greatwall from mitotic extracts led to the accumulation of inhibitory phosphorylations on Cdc2 kinase. As Greatwall depletion would also prevent cycling extracts from entering M phase, and this could be rescued by constitutively active Cdk1, they concluded that Greatwall participates in the autoregulatory activation loop for Cdk1. This notion found support from experiments in which they showed that activated Greatwall could induce phosphorylations of Cdc25 in the absence of the activity of kinases of the activation loop or in the presence of an activator of protein kinase A that normally blocks mitotic entry [44]. These effects are all very similar to those of the phosphatase inhibitor okadaic acid, and indeed okadaic acid could drive cycling extracts into M phase in the absence of Greatwall. This led them to the idea that Greatwall negatively regulates a crucial phosphatase that inhibits Cdc25 activation and M phase entry.

Meanwhile, the party was joined by a group in Montpellier, originally assembled by Marcel Dorée and with considerable expertise in biochemical studies on Xenopus egg extracts. This group, now led by Anna Castro and Thierry Lorca, showed that depletion of Greatwall also promoted mitotic exit, even in the presence of a high Cdk1 activity, by inducing dephosphorylation of mitotic substrates [39]. Two findings led to the idea that Greatwall activity was inhibiting PP2A. First, the depletion of PP2A from mitotic extracts rescued the phenotype induced by loss of Greatwall; and second, the PP2A-dependent dephosphorylation of Cdk1-cycB substrates was increased in Greatwall-depleted *Xenopus* egg extracts. This idea that Greatwall inactivates ‘antimitotic’ phosphatases also found support from the Goldberg group [40], who showed that, once activated, Gwl promotes inhibition of the PP2A trimer with the B55δ subunit (counterpart of B55^twins^ of *Drosophila*). In the absence of Greatwall, PP2A-B55δ remained active even when Cdk1 activity was high. Moreover, the removal of PP2A-B55δ corrected the inability of Greatwall-depleted extracts to enter M phase. Thus, there appeared to be two components to Greatwall function: one to inhibit PP2A to promote the Cdk1 activation loop and a second to suppress the PP2A activity that would otherwise remove Cdk1-driven phosphorylations [45]. Thus, there is some ambiguity in interpreting the requirements for Greatwall in inhibiting PP2A; some experiments emphasized its role to promote in mitotic entry and others to maintain the mitotic state.

**Figure 4.**
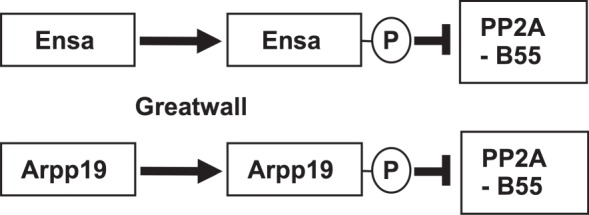
The paralogues Ensa (Endos) and Arpp19 are phosphorylated by Greatwall kinase to become inhibitors of PP2A-B55.

Clarity into the biochemical mode of action of Greatwall kinase came from the identification of its principal substrates [41,42]. Tim Hunt's laboratory had been studying the roles of protein phosphatases in mitotic progression for some time and also had convincing evidence that in *Xenopus*, PP2A-B55δ was indeed the major phosphatase for Cdk1 substrates; depletion of this form of PP2A accelerated mitotic progression in mitoic extracts [46]. As they were not able to detect phosphorylated forms of PP2A, they were led to suspect a role for Greatwall in phosphorylating some intermediate protein to achieve PP2A-B55δ inhibition. This led them to screen for Greatwall substrates in interphase egg extracts and identify α-endosulphine (Ensa) and a related protein, Arpp-19, a substrate of cyclic AMP-activated protein kinase in post-synaptic neurons [47]. The Montpellier group reached similar findings in screening interphase extracts for Greatwall substrates [42] to find the same two proteins. Once phosphorylated by Greatwall these proteins became inhibitors of PP2A-B55δ. In the absence of Gwl activity, Arpp19 and α-Endosulphine were dephosphorylated, and lost their capacity to bind and inhibit PP2A. The London and Montpellier groups disagree about the relative importance of ARPP19 or Ensa in frogs, but as the two proteins are so highly similar, it may be questionable whether any distinction is of biological importance. Endos, the single orthologue of these proteins in *Drosophila*, is phosphorylated on the equivalent serine residue by the fly Greatwall kinase. Mutations at this site abolish the ability of the protein to rescue Endos depletion in cultured *Drosophila* cells [28]*.* Thus, genetic and biochemical approaches converged to identify this novel form of mitotic regulation by a protein kinase. Greatwall promotes mitotic progression not by phosphorylating a particular protein to directly promote its mitotic activity but rather to enable the inhibitor of an anti-mitotic phosphatase (figure 4).

**Figure 5.**
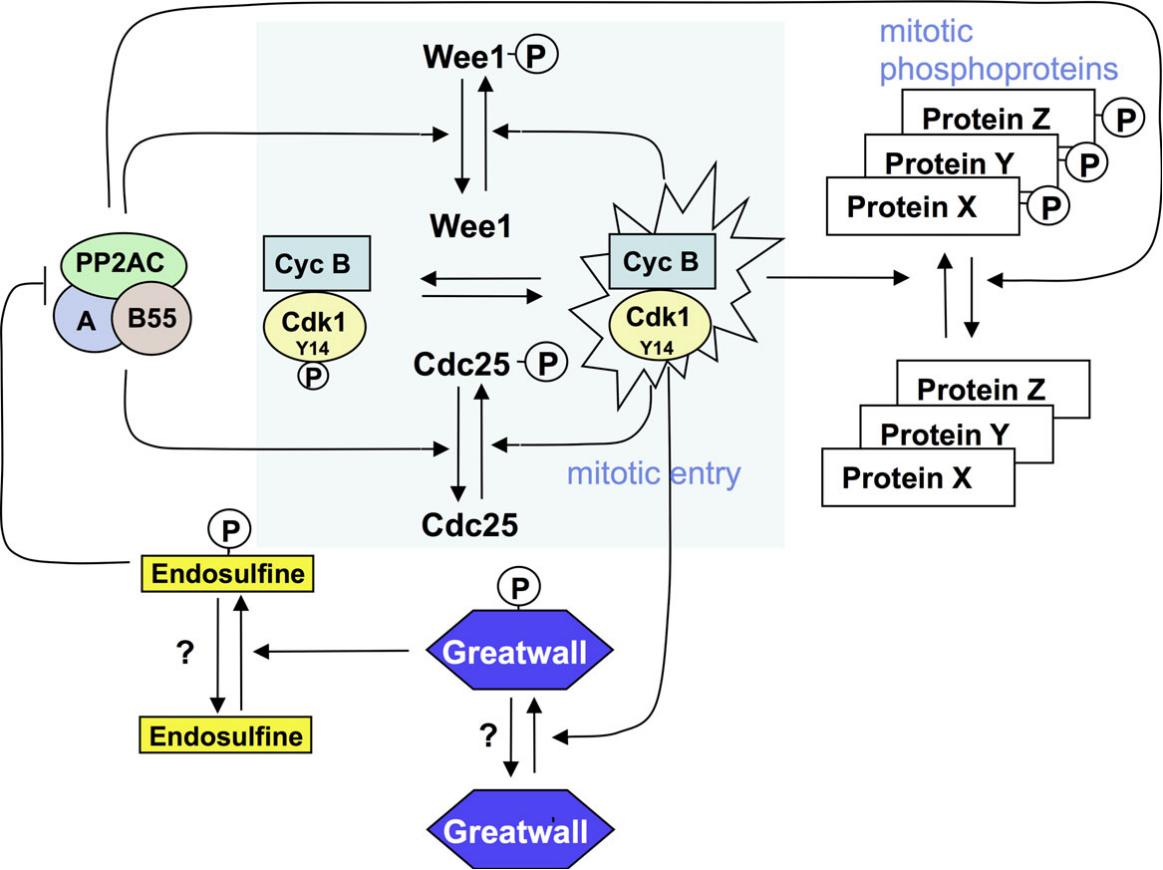
Greatwall–Endos regulates mitotic entry and stabilizes the mitotic state by inhibiting PP2A-B55. Mitotic entry is regulated by a positive-amplification loop in which the dual-specificity phosphatase Cdc25 dephosphorylates and thereby activates Cdk1-cycB kinase. Cdk1-cycB phosphorylates and activates Cdc25. Cdc25 is opposed by the Wee1 kinase that is inhibited by Cdk1-cycB phosphorylation. Thus, PP2A dephosphorylates Cdc25-P and Wee1-P to oppose Cdk1-cycB. This accounts for the long-known fact that inhibition of PP2A (by okadaic acid) promotes mitotic entry. Endos, phosphorylated by Greatwall, acts in an analogous way. By inhibiting PP2A, phospho-Endos also maintains the mitotic state by enabling the multiple mitotic substrates of Cdk1-cycB to retain their phosphate groups.

Aside from their importance in opposing the activity of PP2A to reverse the phosphorylation of Cdk1 substrates in mitotic exit, Greatwall kinase and its Endos substrate now emerge as key components of the regulatory loop that governs mitotic entry. This is because PP2A inhibition results in the accumulation of the phosphorylated, active form of Cdc25 and the phosphorylated, inhibited form of the Wee1 kinases. The consequence—full activation of Cdk1 even at low levels of Cyclin B—accounts for the long-standing observation that the phosphatase inhibitor okadaic acid can promote mitotic entry [48]. This has led Bela Novak and has collaborators to point out that the inhibition of PP2a by Greatwall/Endos contributes additional amplification loops to an inherently bistable mitotic switch that governs mitotic entry. This reflects the antagonistic interaction between one group of proteins promoting M phase (Cdk1-cyclin B, Cdc25, Gwl and Endos) and another that promotes interphase (Wee1, PP2A-B55; figure 5) [49,50]. As might be expected, such a central tenet for the regulation of mitotic entry is highly conserved, and the human counterpart of Greatwall, MASTL kinase (microtubule-associated serine threonine kinase-like protein), has similar roles in mitotic progression [51–53]. Indeed, the mitotic defects resulting from the depletion of MASTL can be rescued by simultaneous knockdown of PP2A or treatment with okadaic acid, once again indicating the importance of regulating the balance between Cdk1 and PP2A activity [51]. Interestingly, downregulation of MASTL can even overcome the mitotic arrest resulting from failure to activate the anaphase-promoting complex/cyclosome (APC/C) in cells ablated for Cdc20 [53]. Thus, by relieving the inhibition of PP2A-B55, it is possible to overcome even this block to the natural progression through the metaphase–anaphase transition and exit mitosis.

## 5. Greatwall antagonizes some key functions of Polo

In spite of the clarity of understanding we now have of this regulatory loop, the relationship between Polo and Greatwall functions is confusing. Paradoxically, both Polo and Greatwall kinases promote progression through mitosis, and yet the genetic interactions outlined above suggest that the gain-of-function mutation *gwl^Scant^* negatively regulates the function of Polo or one of its targets. So what is the evidence that the mitotic kinases Polo and Greatwall can act antagonistically, and how can we account for this? In considering this conundrum, it is important to note that this antagonism is observed specifically with respect to what appears to be a sensitive threshold requirement for Polo kinase activity to maintain centrosomes at the nuclear envelope in the division cycles of syncytial embryos. This phenotype can also be seen in other situations in which Polo kinase activity is reduced; for example, following the over-expression of Map205, a known interphase-binding partner of Polo that sequesters the kinase onto microtubules [54].

Two other lines of evidence suggest that the *polo^1^+/+ Scant* phenotype represents an enhancement of the *polo* phenotype as a result of the gain-of-function mutation in the Greatwall kinase. First, *polo^1^+/+ Scant* maternal effect lethality can be rescued by increasing the activity of Polo kinase, for example, as a result of *polo^+^* duplications we obtained in the screen for revertants [27]. Second, the degree of embryo lethality resulting from the cumulative effects of centrosome loss covaries with strength of *polo* allele. The weak hypomorphic allele, *polo^1^*, shows only moderate embryonic defects with *Scant*, whereas the amorphic allele, *polo^11^*, shows centrosome loss defects that prevent any embryonic survival. Because the function of *Scant* can be ascribed to a mutation that we demonstrated to result in hyperactive Greatwall kinase [27], these experiments suggest either that Greatwall kinase might decrease the level of active Polo via PP2A or that Greatwall is independently inhibiting a pathway that is positively regulated by Polo.

Further evidence supporting a role for Greatwall in antagonising Polo has come from a recent study from Daniela Drummond-Barbosa's laboratory to search for second-site non-complementing mutants of *endos* [55]. Mutation in *matrimony* (*mtrm*), which encodes a known Polo kinase inhibitor [56], resulted in mitotic abnormalities in syncytial embryos when transheterozygous with an *endos* mutant in mothers (i.e. *mtrm +/+ endos* females). This sterility could be rescued by removing one wild-type copy of *polo*. Thus, in the absence of sufficient Matrimony protein to depress Polo activity, 50 per cent of functional Endos is unable to correctly exert mitotic control over a Polo-regulated function. These observations are thus consistent with the Greatwall–Endos pathway negatively regulating Polo.

**Figure 6.**
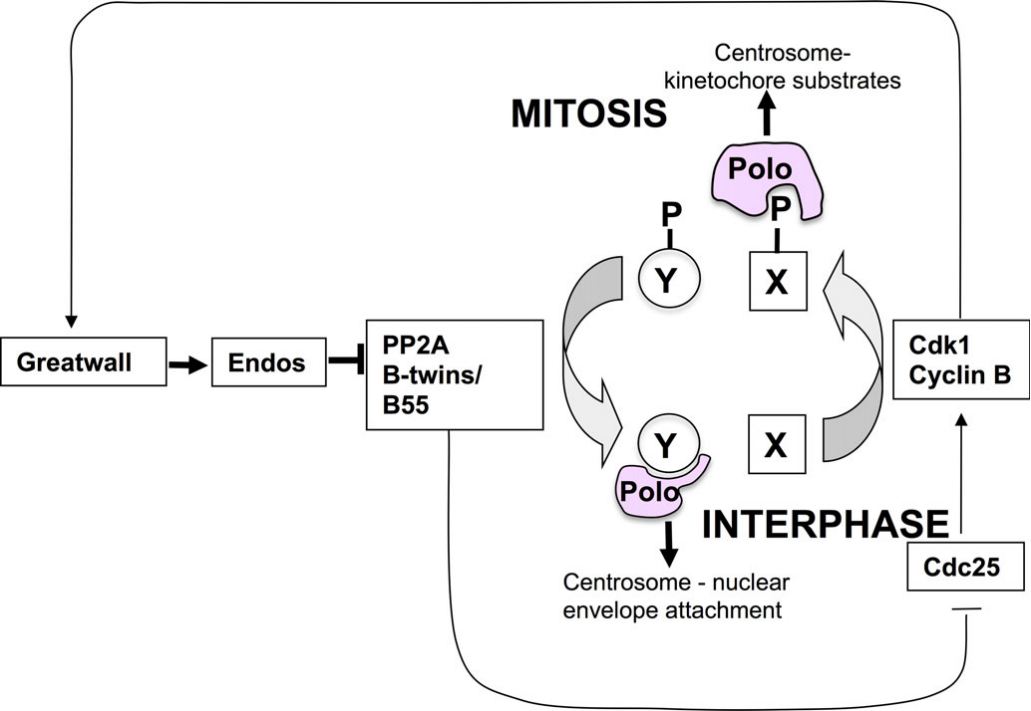
Hypothesis for how Greatwall might act antagonistically to Polo late in mitosis in the syncytial nuclear division cycles of the *Drosophila* embryo. As both Greatwall and Polo are ‘mitotic kinases’, it seems counterintuitive that Greatwall might inhibit some Polo functions as suggested by the interactions between the *gwl^Scant^* and *polo* mutations. Several explanations for this are possible and are discussed in the text. This schematic presents one of these potential explanations. It postulates that because Polo can interact in mitosis with proteins (X-P) that have been phosphorylated by Cdk1-cycB, and at mitotic exit and interphase with proteins that do not have such mitotic phosphorylations (Y), downregulating PP2A in Greatwall-Scant-derived embryos can prolong Polo's interactions with its mitotic partners and deny its interactions with interphase partners. In the context of this scheme, the consequence of the latter would be to favour retention of high Y-P levels and thereby lead to loss of centrosomes from nuclei, postulated to be an interphase process requiring dephosphorylated protein Y.

There are a number of possible ways to account for this negative regulatory relationship between Greatwall–Endos and Polo that need not necessarily be exclusive and certainly still need to be clarified. First, both Polo and PP2A have been shown to be required for the centrosome maturation [57], and thus by promoting inhibition of PP2A, Greatwall would essentially antagonize a pathway promoted by Polo. Second, it has been proposed that loss of PP2A function synergizes with loss of Polo function because both activities are required to maintain the association of centrosomes to the nucleus or spindle, albeit at different stages of the nuclear division cycle [58]. A third possibility is that at some stages of the cycle, Greatwall and Polo together promote mitotic progression, whereas at other stages they act antagonistically. This is certainly possible because of the multiple ways in which Polo can interact, through its Polo-box domains, with partner proteins. The Polo-box domain generally binds phosphorylated sequences on a partner protein that has either been primed by another kinase or self-primed by Polo itself [58]. Thus, as the cell cycle proceeds we see a progression from Polo interactions at mitotic exit and in interphase that can be not only independent of Cdk1 but can also be actively disrupted by Cdk1-cycB phosphorylation to ones from late G2 until anaphase that are totally dependent on priming phosphorylation by Cdk1-cycB (box 2). We have argued that it is the alternation of Polo's functional interactions between a Cdk1-cycB dependency and independency that might account for the paradoxical relationship between Greatwall and Polo evidenced by centrosome detachment in the syncytial cycles of *polo +/+ Scant-*derived embryos [27]. We proposed that in prophase and prometaphase Greatwall, activated by Cdk1-cycB, inhibits PP2A via Endos, and this sustains the association of Polo with its Cdk1-cycB-phosphorylated partners. Once cyclin B is degraded at the onset of anaphase, Cdk1 activity falls and Polo begins to associate with proteins dephosphorylated at their Cdk1 sites by PP2A-B55^Twins^. We postulate at least one such of these latter proteins, which undergo Cdk1 independent interactions with Polo, to be required for the maintenance of centrosome attachment to the nuclear envelope. In syncytial embryos, hyperactive Greatwall^Scant^ kinase would lead to reduced interphase activity of PP2A, so lowering levels of functional complexes between Polo and dephosphorylated partners below some critical level (figure 6). At present, however, we do not know the molecular players participating in this process, nor indeed whether it reflects a single molecular interaction or a rather a readout of the effect of disrupting the Cdk1-PP2A balance on this stage of mitotic progression.

Box 2. **Diverse interactions of the Polo-box domain of Polo/Plk1 kinases with their partner proteins.**

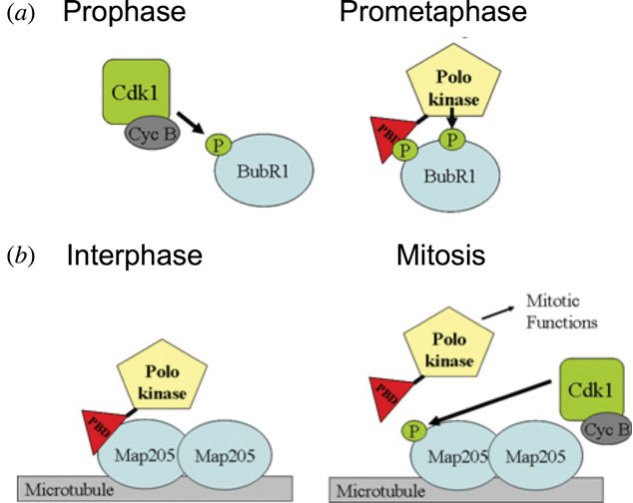

The C-terminal part of the polo-like kinases has two Polo-box motifs that form interaction sites with partner proteins. Typically, a priming phosphorylation on the partner protein mediated by another protein kinase generates a docking site for Polo/Plk1. (*a*) In mitosis, the priming kinase is often Cdk1-cycB itself, thus ensuring that targeting of Polo to specific sequences occurs only when mitosis is underway. The binding of Plk1 to, and its subsequent phosphorylation of, the checkpoint protein BubR1 exemplifies such an interaction, likely to mediate the association of Plk1 with the kinetochore [59–61]. (*b*) Plk1 also interacts with other partners when Cdk1 is inactivated following cyclin B degradation. Perhaps the best examples of these interactors and substrates are the microtubule PRC1 protein [62] and the central-spindlin subunit CYK-4 that each participate in mediating Polo functions in the early stages of cytokinesis [63,64]. It is postulated that the initial phase of binding of Plk1 to such proteins may be phosphorylation-independent, but that subsequent phsophorylation of the target protein by Plk1 may effectively act as a self-priming event and accentuate the interaction [65]. We recently described an extreme case of an interaction of this type in syncytial *Drosophila* embryos, where a microtubule-associated protein Map205 sequesters Polo kinase onto microtubules during interphase (shown here). This interaction, which also takes place via the Polo-box domain, is actually disrupted by Cdk1-cycB phosphorylation of Map205 at an adjacent site (figure 6*b*) [54].

## 6. Regulation of Greatwall activity

Although available evidence supports the idea that Greatwall is activated by Cdk1, the precise mechanism for this is not clear and the possibility still exists that other mechanisms are involved. Greatwall is a member of the AGC family of kinases that includes enzymes such as PKA, PKC and RSK. Typically, the activation of this group of enzymes requires phosphorylation of an activation loop in the C-lobe of the enzyme, together with interactions between N- and C-terminal tails. The latter interaction involves phosphorylation of a hydrophobic motif that appears to be absent from Greatwall and it has been suggested that another AGC kinase might interact with Greatwall to provide this [66]. Much remains to be done in order to understand how exactly the activity of Greatwall is regulated. Greatwall is activated by Cdk1 or a Cdk1-dependent protein kinase, but although Greatwall's phosphorylation sites have been mapped [66], the significance of each site needs further investigation. Equally little is known of why the K97M mutation results in the activation of Greatwall^Scant^ in *Drosophila* [27]*,* although introducing the equivalent mutation into *Xenopus* Greatwall kinase (K71M) also results in a hyperactive enzyme [67]. In fact, this mutant form of Greatwall is able to induce oocytes to enter M phase even in the absence of progesterone, the normal hormonal stimulus for this process. We are even more ignorant of the protein phosphatases required to inactivate Greatwall on mitotic exit, or indeed to inactivate its substrate Endos. It has, however, been suggested that Endos might be dephosphorylated at mitotic exit by PP1 [50]. If so, this may contribute to another interesting regulatory loop because PP1 has itself been shown to be inhibited by Cdk1s [68].

## 7. What other roles might Greatwall have?

While the focus has naturally been upon the role of Greatwall in mitosis, other studies raise the possibility of its role in different processes. It has been reported, for example, that Greatwall promotes recovery from the DNA damage checkpoint [69,70]. Thus, an increased DNA damage response was seen in *Xenopus* extracts depleted of Greatwall, whereas active Greatwall kinase inhibited the response. Greatwall was itself inhibited by the DNA damage response in a caffeine-sensitive manner, indicating a response to ATM (ataxia telangiectasia mutated)/ATR (ATM-related) signalling. The mechanisms of neither this inhibitory effect nor of the interphase activation of Greatwall in response to DNA damage are clear. However, it is of interest that Greatwall and Plk1 appear to associate and that the two kinases appear to show mutual dependency in promoting recovery from the damage checkpoint. Plk1 appears able to phosphorylate Greatwall directly, whereas it is suggested that Greatwall activates Aurora A, which in turn activates Plk1 [70]. These interactions and the precise roles of the phosphorylation reactions resulting from them demand more detailed study in different systems before we have a true understanding of Greatwall activation both in the damage checkpoint recovery and in mitotic entry.

We might also expect Greatwall and Endos or their counterparts to function outside of the cell cycle given that PP2A-B55^twins^ functions in a wide range of biological processes. In *Drosophila*, there is evidence for involvement of the *twins* gene in the maintenance of neuroblast polarity, pattern formation in imaginal discs and in sensory organ development. Thus, as a PP2A-B55^twins^ inhibitor, Endos could also participate in these processes. Moreover, it also seems that the Endos family of proteins might have other functions beyond the regulation of PP2A-B55. The budding yeast counterpart of Greatwall and Endos, the respective Rim5 protein kinase and its substrates Igo1 and Igo2, participate in the response to limiting amounts of nitrogen and/or carbon sources. Such starvation leads to downregulation of the conserved TORC1 and PKA signalling pathways and the consequential activation of Rim15 kinase, which in turn controls expression of specific downstream genes by regulating both transcription and mRNA stability. Once phosphorylated by Rim15, the Igo1 and Igo2 proteins associate with the mRNA decapping activator Dhh1 and protect newly expressed mRNAs from the mRNA decay pathway [71]. If these results extend into analogous pathways in higher eukaryotes, then this could identify a whole new range of functions for the Greatwall kinase. The participation of Arpp19 in stabilizing GAP-43 mRNA in response to nerve growth factor treatment could perhaps turn out to work through a similar mechanism [72]. Indeed, there are also some hints that Endos might be involved in mRNA stability, which come from the finding indicated above that in females heteroallelic or hemizygous for *endos*, where its protein levels are reduced by more than 95 per cent, levels of Polo and Cdc25^twine^ protein are drastically reduced [31]. Thus, Endos could have an additional role in the post-transcriptional regulation of gene expression leading up to meiosis in the female germ-line of *Drosophila.* What is undoubtedly clear is that it is truly difficult to study protein phosphatase functions because of the need to relate them to those of the counteracting protein kinase. If the protein phosphatase regulatory proteins are as pleiotropic as it seems, then we must look forward to some very interesting times.
